# Pandemic Disruptions to Private Pathology Testing Uptake in Australia, 2019–2024

**DOI:** 10.3390/pathogens14080812

**Published:** 2025-08-15

**Authors:** Melanie Keech, Shane Kavanagh, John Crothers, Liliana Orellana, Catherine M. Bennett

**Affiliations:** 1 School of Health & Social Development, Faculty of Health, Deakin University, Waurn Ponds, Geelong, VIC 3216, Australiacatherine.bennett@deakin.edu.au (C.M.B.); 2 Institute for Health Transformation, Faculty of Health, Deakin University, Waurn Ponds, Geelong, VIC 3216, Australia; 3 Pathology Awareness Australia, Sydney, NSW 2065, Australia; john@enablement.au; 4 Biostatistics Unit, Faculty of Health, Deakin University, Waurn Ponds, Geelong, VIC 3216, Australia; l.orellana@deakin.edu.au

**Keywords:** pathology, cancer, COVID-19, telehealth, diagnosis

## Abstract

A new human pathogen triggering a pandemic can impact health directly through disease resulting from infection and indirectly through health system disruption. The COVID-19 pandemic is hypothesised to have impacted pathology testing by impacting healthcare and pathology operations and reducing healthcare attendance for fear of infection. The impacts of COVID-19 incidence and pandemic control measures on non-COVID pathology testing were assessed in four Australian states/territories using pathology data (histology, prostate-specific antigen, gynaecological cytology, complete blood count, haemoglobin A1c, and human immunodeficiency virus) from a large national private pathology provider (January 2019–December 2024). Weekly testing volumes from lockdown periods were compared to the equivalent weeks in 2019. All pathology tests demonstrated a substantial decline during the initial national lockdown in March 2020. Subsequent lockdowns were also associated with disruption. For example, complete blood count testing in Victoria was −22% in March 2020 and −5% in the second wave that year. Total annual testing volumes were lower for all tests in 2020 compared to 2019, excluding haemoglobin A1c, and reduced testing persisted through to 2024. The findings indicate substantial and sustained negative pandemic impacts on pathology testing. Reductions in pathology testing signal heightened risk of delayed disease diagnosis, disrupted chronic disease management, and poorer health outcomes.

## 1. Introduction

Pathology testing is integral to healthcare enabling diagnosis, prevention, monitoring, and treatment of disease [1]. Missed tests can result in delayed or missed diagnosis of communicable diseases, and inadequate chronic disease management, delayed diagnosis and consequently increased morbidity and mortality [2,3]. This is particularly the case for faster-growing cancers, where short delays in diagnosis can result in significant reductions in health outcomes and life expectancy [2].

International evidence suggests the COVID-19 pandemic disrupted pathology testing with an average reduction of 45.3% in cytological samples observed across 23 countries during the first four weeks of lockdown in 2020 [4]. In the Netherlands, the first lockdown saw reductions in histological and cytological samples, with smaller reductions in the second lockdown. For example, gallbladder samples experienced a 71% reduction in the first lockdown and 19–24% reduction in the second lockdown [5].

There is also evidence of sustained impacts. A US study found reductions of 29% for Papanicolaou smears and 16% for haemoglobin A1c (HbA1c) four months after lockdown was lifted [6]. The Netherland’s study reported only 86% and 92% of the expected annual volume of histological and cytological samples respectively by the end of 2020 [5].

In Australia, multiple pandemic-related factors may have impacted pathology testing. Strict disease control measures were implemented to limit transmission, including state-wide lockdowns, particularly in Victoria (Vic) and New South Wales (NSW) [7]. These measures were in place for extended periods throughout 2020 and 2021 [7]. While access to medical care or pathology testing was not formally restricted, elective surgeries were reduced, and staff shortages along with physical distancing requirements limited clinic and pathology collection centre capacity [7]. Public confusion regarding permitted reasons for leaving home during lockdowns would have also contributed [8]. Heightened concern about exposure to SARS-CoV-2 in medical and pathology settings and changes in service provision, like increased telehealth access, may have affected test completion rates [9].

Despite being amongst the countries with the most stringent pandemic response in place, studies on how the pandemic influenced pathology testing rates in Australia are limited. One study from South Australia examined the impact of lockdown periods in 2020 on testing rates, suggesting a 47% reduction in haematology, endocrinology and general chemistry pathology tests compared to a pre-lockdown period in 2020 [10]. Similarly, a self-published report from a national pathology company indicated reductions in histopathology, HbA1c and cholesterol testing between January and October 2020 [11].

This study assesses the short- and longer-term impacts of the pandemic and related disease control measures on non-COVID pathology testing in Australia by comparing testing volume trends from a large private pathology provider across jurisdictions subjected to different restriction measures. It also examines whether the introduction of telehealth services helped mitigate disruptions to healthcare access during this period.

## 2. Materials and Methods

### 2.1. Data

This study used non-COVID pathology testing data routinely collected by a large private pathology provider from 1 January 2019 to 29 December 2024, for Vic and NSW, the two most populated states that also experienced the largest outbreaks and lockdowns of longest duration, and for comparative purposes, Queensland (Qld), and Western Australia and the Northern Territory combined (WA/NT). The first true community-wide SARS-CoV-2 transmission was not experienced until January 2022 with the reopening of Australia’s international borders coinciding with the arrival of omicron BA.1, though this was delayed a further two months in WA with state borders not reopening until 3 March 2022 [12].

The provider’s identity has been anonymised due to market sensitivities. However, it is one of the top three laboratory networks serving a representative and substantial segment of the community Australia-wide, with services covering a range of urban and rural areas across multiple states and territories in Australia. Using provider data overcomes limitations with publicly available Medicare Benefits Schedule (MBS) data which represents services subsidised by the Australian Government. MBS data do not include public hospital services to public patients [13]. Importantly, MBS data are also impacted by episode coning where only the three most expensive tests ordered by a General Practice (GP) are billed to Medicare, leaving cheaper and common tests unrecorded [14].

Six pathology tests were selected for inclusion in the study. The choice of tests was made after consultation with the pathology provider to select a sub-set of pathology tests most likely to capture overall health service functioning and potential pandemic impacts including across pathology tests associated with cancer, chronic and infectious disease. These were complete blood count (CBC), histology, prostate specific antigen (PSA), gynaecological cytology (HPV), human immunodeficiency virus (HIV) and HbA1c. For HbA1c, total tests were examined as well as total counts for MBS item numbers 66551, used for monitoring diabetics, and 66841 used for diagnosing diabetes. The purpose of tests is included in the test request.

All tests completed by the provider across metro and regional services, and hospital and community centres for each state/territory were summarised as daily average volume by week or month. Averages were calculated over 5 day working week, including public holidays that fall on usual working days. The ratio of metro to regional laboratories was consistent between the states/territories, while the distribution of community to hospital-based services varied by state/territories. There were no changes in provider catchment throughout the study period that would influence testing volume, and, as the data are required for billing processes, there is unlikely to have been under-reporting during high demand periods.

In addition to the private provider data, quarterly counts of HIV Antibody rapid test sales data were acquired from a commercial medical supplies company for 2019–2022 to examine whether fluctuations in HIV laboratory testing were offset by changes in rapid test use. Weekly COVID-19 case numbers were obtained from a public dataset, collating data from the federal, state and territory health departments [15]. Monthly GP service utilisation was identified from public MBS data [16] using face-to-face GP consults including item numbers: 3, 23, 36 and 44 and telehealth: 91790, 91800, 91801, 91802, 91795, 91809, 91810, 91811, 91890 and 91891 [17,18].

A week was classified as a lockdown period if at least one day of the week the state/territory was in lockdown. Where state-level policies varied between metropolitan and regional areas, lockdown periods were based on metropolitan lockdowns as these areas contribute the majority of testing for the state. Given staged easing of lockdown restrictions, the end of a lockdown was coded as the day before non-essential businesses opened for trading in some face-to-face capacity. Lockdowns were only counted if they lasted two weeks or more given a greater likelihood of measurable impacts on pathology services. The fifth and sixth Vic lockdowns were treated as a single period given only 9 days separated them. Lockdown periods are outlined in Table 1.

### 2.2. Data Analysis

Analysis was descriptive, no statistical tests were conducted as data are at the “population” level for one large private pathology provider that can’t be consider “representative” of the volume of pathology tests conducted for relevant states/territories in Australia. All results are presented at the state/territory level. Initially, daily average tests by week were plotted alongside pandemic public health measures and case numbers for the period of 2019–2024. For each lockdown period (longer than 14 days) we then calculated the percentage difference relative to the same calendar period in 2019 for each test, except HPV (see Table 1). We further examined changes in daily average tests by week during selected lockdown periods (second and fifth/sixth combined for Vic and the second lockdown for NSW) across corresponding calendar periods relevant for the acute phase (2019–2022). This analysis was undertaken for histology and CBC, which are two common tests with broad application and indicative of overall testing impacts. For each calendar year smooth estimates and 95% confidence intervals were obtained under linear mixed models including cubic splines with four user-defined knots (Stata commands mkspline and MIXED). Monthly daily average of test numbers for CBC for each state/territory were plotted alongside lockdown periods, face-to-face, telehealth, and total GP visits for the acute phase (2019–2022). For each state/territory, we present: (a) plots of the weekly percentage difference in cumulative test numbers relative to 2019 for CBC and histology; and (b) the percentage difference in the total number of tests for each year compared to 2019 for all tests. Data were analysed using Microsoft Excel v16.99.2 (Microsoft Corp., Redmond, WA, USA) and StataSE v17 (StataCorp LLC, College Station, TX, USA) software.

Ethics exemption was obtained from the Deakin University Human Research Ethics Committee (2022-014).

## 3. Results

Across the jurisdictions for the analysis period, daily average test counts were highest for CBC, followed by HbA1c, histology, PSA, HIV and HPV. While total testing numbers for each test varied by year, the proportion of each to the total number of tests in a year remained consistent, except for HPV which was more common than PSA and HIV in 2019 and contributed a smaller proportion in later years. This coincides with the progressive introduction of new test protocols with the change to a five-year interval with the Cervical Screening Test rather than the previous two-year interval between Pap tests [19]. The largest overall number of tests for this pathology provider were reported in Vic, followed by Qld, NSW and WA/NT.

### 3.1. Daily Average Tests

**Figure 1:**
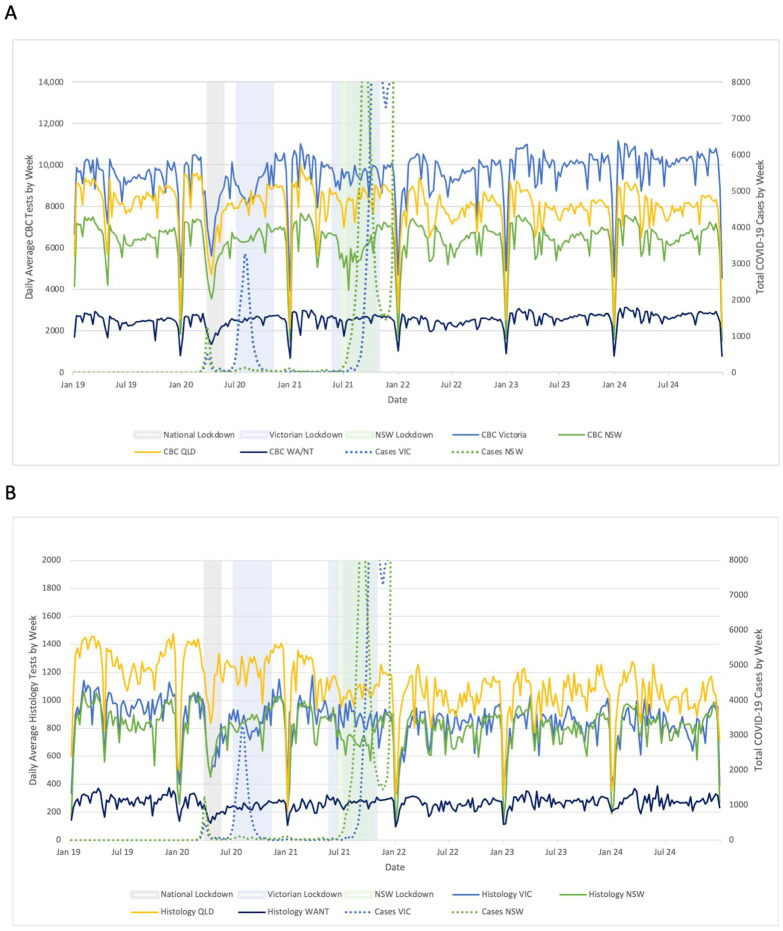
(**A**) Daily average CBC tests by week, versus lockdown periods and COVID-19 case numbers, by state/territory; (**B**) Daily average histology tests by week, versus lockdown periods and COVID-19 case numbers, by state/territory. Case numbers were not included after the start of May 2022 due to sustained high cases numbers which could not be appropriately graphed.

Reductions in weekly average test numbers occurred during weeks with public holidays. Substantial reductions were also apparent during the initial national lockdown period from 23rd March until 20th of May 2020 with a drop in counts across all tests, larger and sustained for longer than would normally be expected at Easter. The reductions were smaller in magnitude over subsequent lockdowns (Figure 1, Table 1).

**Figure 2:**
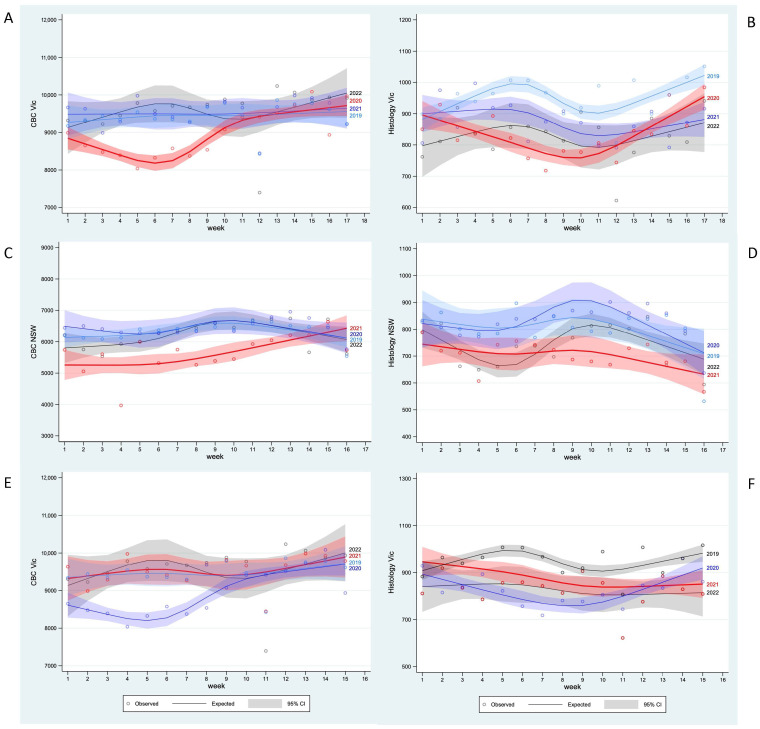
Observed values and cubic splines estimated (95% CI) daily average test counts by week in the acute pandemic phase for: (**A**) CBC during the second Vic lockdown in 2020 (red), compared to the corresponding calendar periods in 2019, 2021 and 2022; (**B**) histology counts by week during the second Vic lockdown in 2020 (red), compared to the corresponding calendar periods in 2019, 2021 and 2022; (**C**) CBC counts by week during the second NSW lockdown in 2021 (red), compared to the corresponding calendar periods in 2019, 2020 and 2022; (**D**) histology counts by week during the second NSW lockdown in 2021 (red), compared to the corresponding calendar periods in 2019, 2020 and 2022; (**E**) CBC counts by week during the fifth and sixth Vic lockdown in 2021 (red), compared to the corresponding calendar periods in 2019, 2020 and 2022; (**F**) histology counts by week during the fifth and sixth Vic lockdown in 2021 (red), compared to the corresponding calendar periods in 2019, 2020 and 2022.

Reduced testing that commenced soon after the second Vic lockdown in 2020 was imposed was followed by a recovery later in the lockdown period (Figure 2A,B). In comparison, for the second NSW lockdown, recovery was only visible for CBC (Figure 2C,D). No pattern was apparent during the fifth and sixth Vic lockdowns (Figure 2E,F).

Reductions in test volume aligned with increases in COVID-19 case numbers during the national and second Vic lockdowns. This relationship was reduced throughout the later Delta and Omicron waves (Figure 1).

### 3.2. GP Consultations

**Figure 3:**
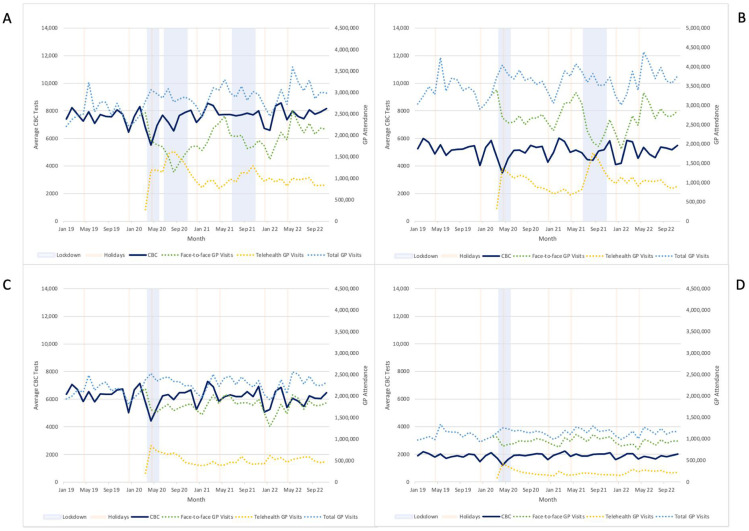
Daily average CBC tests by month versus face-to-face, telehealth, and total GP attendances by state/territory (**A**) Vic; (**B**) NSW; (**C**) QLD; (**D**) WA/NT.

As expected, telehealth GP consultations were inversely related to face-to-face GP attendances during lockdowns, and recovery in testing numbers corresponded with peaks in telehealth during lockdowns (Figure 3).

### 3.3. Cumulative Testing Numbers

**Figure 4:**
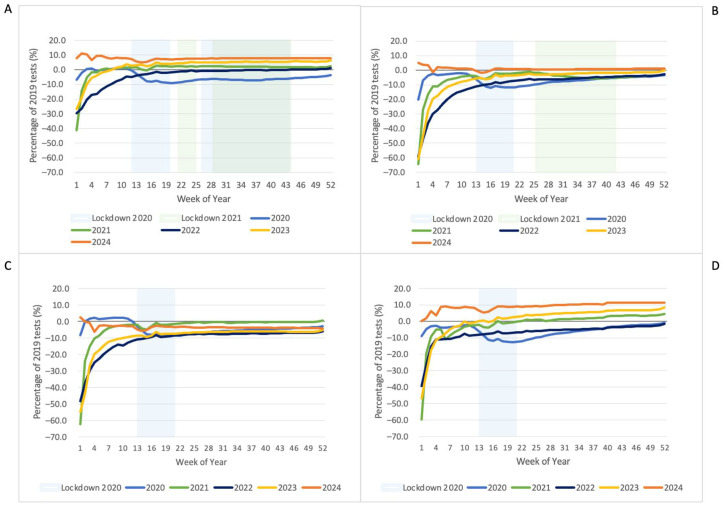
Percentage difference of cumulative CBC tests relative to 2019 by year and state/territory: (**A**) Vic; (**B**) NSW; (**C**) QLD; (**D**) WA/NT.

**Figure 5:**
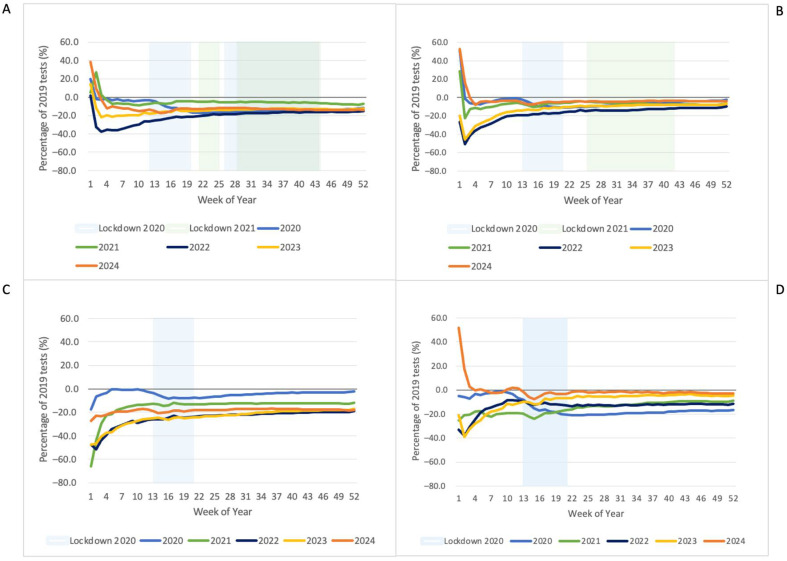
Percentage difference of cumulative histology tests relative to 2019 by year and state/territory: (**A**) Vic; (**B**) NSW; (**C**) QLD; (**D**) WA/NT.

Figure 4 and Figure 5 present the percentage difference in cumulative tests over time compared to 2019. Despite some increases in testing after lockdowns, in many instances the increase did not fully compensate for the reduction during lockdowns. The high variability in the earlier weeks is a product of lower numbers and the impact of varying holiday dates.

All states/territories displayed a sustained reduction across all tests in 2020 relative to 2019, except HbA1c. In 2021 and 2022, reductions were variable by test and state/territory, with reductions persisting for some tests for the entire study period (Table 2).

In addition to reductions in HIV pathology tests (Table 2), rapid HIV tests also showed large reductions for 2020–2022 (Table 3), suggesting that reductions in HIV pathology testing were not compensated by the alternative testing method.

## 4. Discussion

The pathology testing trends during the acute pandemic phase were complex and varied by test and jurisdiction. Lockdowns and increases in reported COVID-19 case numbers were coincident with decreased testing in the initial stages of the pandemic for some, but not all tests. Of note is evidence of reductions in testing extending through until the end of 2024 for some tests and in some states/territories. Of particular concern are reductions in histology testing across all states/territories of between 2.2–19.0% for the study period.

Testing patterns over time need to be contextualised against an expectation of natural growth in testing, driven by population growth, an ageing population, increased disease incidence and changes in testing practices. The average growth in Medicare claimed pathology tests was 3.5% per year from 2003–2018 [1,13], but exact growth varies by test, year, and state/territory. It is also unclear what level of growth occurred within jurisdictions given substantial reductions in net overseas migration in 2020 and 2021, and then subsequent increases in net overseas migration in 2022–2024 [20]. Nevertheless, the results are indicative of disruptions in the way Australians interacted with the healthcare system.

Even though the most substantial reductions in pathology testing occurred during lockdowns, it is difficult to determine the individual contributions of the lockdowns themselves to this pattern. Other factors may have also been influential in reducing testing numbers. The lockdowns coincided with higher COVID-19 case numbers and there may have been reduced clinic and collection centre capacity given diversion to COVID-19 patients and testing. In addition, staff absences due to illness or infection control measures may have reduced capacity [7].

However, several findings indicate the role of risk perception of the public and healthcare industry. Firstly, despite no restriction prohibiting Australians from accessing pathology services during lockdowns, there were reductions in testing of between 15.6–35.9% in the national lockdown and clear reductions for histology and CBC during the second Vic lockdown. It is possible that fear of contracting COVID-19 [21] whilst at health care facilities or pathology collection centres was a factor [11]. The reduced disruption seen in later lockdowns may be attributable to clearer messaging about medical appointment’s being exempt from public health orders, reduced fear in general, better public health messaging about relative risks, vaccinations, and the effects of the introduction and increased utilisation of telehealth [22].

NSW experienced a drop in testing prior to their second lockdown which may indicate anticipation of policy change, but it may also reflect recognition of the rising underlying COVID-19 exposure risk as opposed to the policy change itself. This is in keeping with the contrasting larger reduction observed during the second Vic lockdown a year earlier, and then the lack of change during the fifth and sixth Vic lockdowns, though at a similar time of year, when compliance and concern appeared to have waned [23].

The findings of this study support previous research indicating reduced testing during lockdowns, slow recovery for some tests [5,6], reduced impacts during subsequent lockdowns, and lower overall testing in 2020 [5]. Furthermore, they are consistent with a report from Cancer Australia showing 5.6% fewer diagnostic services in 2020–2022 for MBS items that were sentinel markers of cancer control activity [24] and estimates from the Australian Medical Association of a backlog of 306,281 elective surgery patients nationally at the end of 2021–2022 [25].

The implications of reduced pathology testing vary by type and purpose of test. In the case of HIV, the need for testing varies with sexual behaviour, which itself was likely impacted by lockdowns and restrictions on sex workers and social interaction. There is evidence of shifting patterns of sexual behaviour during the pandemic period in Australia in response to COVID-19 notifications and pandemic restrictions leading to reduced sexual activity [26,27]. For HPV, reductions reflect the change in test and subsequently testing intervals. While for HbA1c, the increase in screening and overall testing numbers likely reflects an ongoing trend of increased use of HbA1c in screening and diagnosis of diabetes [28], along with rising rates of diabetes. Against this background reductions in HbA1c monitoring tests are likely a better indicator of healthcare use and suggest declines in some states/territories and years.

Ongoing reduced histology testing is concerning. Reduced testing may be a result of deferral of non-urgent care, or conversely, be indicative of large-scale delays in cancer diagnosis as a result of an overburdened healthcare system [29], with reduced capacity from increased COVID-19 patients, restrictions on elective surgeries during lockdowns, and exposed and infectious staff isolating. Implications of delayed cancer diagnosis due to these changes are not currently established and will vary by cancer type. However, a 4-week delay in treatment for breast, colon, bladder, and head and neck cancers has been associated with a 6–8% increase in mortality, increasing with further delays [2].

There were several limitations to this study. First, only one year of baseline data were available so historical trends in testing could not be observed. Second, the data were not sensitive to differences in regional and urban pandemic restrictions within states/territories. This may have reduced the detection of lockdown related impacts where they were only applied to urban areas, but as urban residents comprised on average 70% of testing, this is unlikely to impact our ability to detect strong effects. Third, this analysis is based on data from one commercial provider representing only a limited, though substantial and arguably representative subset of all pathology testing. Last, the lack of patient level demographic data limited the ability of the analysis to identify disparities in testing impacts across sub-populations.

A key strength of this study is utilising actual test counts as opposed to MBS billing data. This overcomes concerns with MBS data’s lack of service coverage and missed test counts through data coning, where the recording of only the most expensive tests when tests are batched leads to many individual tests not being captured. As such, this study provides a more accurate insight into pandemic-related impacts on non-COVID pathology testing than those relying on MBS data.

This study provides novel insights using commercial complete pathology test data into the impacts of the COVID-19 pandemic on the functioning of the pathology system in Australia during both the acute pandemic phase and over the longer term. It indicates substantial and sustained negative impacts on pathology testing that suggests the detection and ongoing maintenance of health conditions has been compromised. Further research is required to elucidate changes in testing for population subgroups by age, cultural background, and other demographic characteristics, and according to likelihood of the test result being critical to patient health outcomes. This research could provide insights into social and economic disparities in health service utilisation. In addition, to avoid disruptions of this magnitude in future, we need to better understand the reasons for reduced testing, potential break points in the referral pathway, and the disease burden that has resulted from missed or delayed diagnoses.

## 5. Conclusions

In this study, we found a complex pattern of changes in pathology testing in relation to the pandemic with both shorter term and sustained impacts identified. The findings are a cause for concern given that reductions in pathology testing signal heightened risk of delayed disease diagnosis, disrupted chronic disease management, and poorer health outcomes. This issue warrants further investigation to fully understand the health impacts and to inform future pandemic planning strategies.

## Figures and Tables

**Table 1:** Percentage differences in testing during lockdown relative to same calendar periods in 2019.

			Test				
State/Region	Lockdown No.	Lockdown Dates	CBC	HbA1c (Total)	Histology	HIV	PSA
VIC	1	2020: 23/03–20/05	−22.0	−17.9	−35.9	−35.6	−25.7
NSW	1	2020: 23/03–20/05	−24.7	−17.6	−25.9	−40.3	−32.0
QLD	1	2020: 23/03–20/05	−23.0	−27.7	−15.6	−26.9	−28.7
WA/NT	1	2020: 23/03–20/05	−26.8	−22.0	−39.5	−25.2	−33.1
VIC	2	2020: 09/07–27/10	−5.0	8.2	−12.1	−27.4	−2.7
NSW	2	2021: 26/06–11/10	−10.3	7.7	−12.2	−31.9	−10.6
VIC	4	2021: 27/05–10/06	4.0	18.6	0.1	−20.3	−3.0
VIC	5 & 6	2021: 15/07–21/10	0.7	16.1	−7.5	−23.3	−0.8

Calculation details: Percentage change for row 1 calculated as: (#tests in period for 2020—#tests in corresponding period for 2019)/#tests in corresponding period for 2019. Subsequent rows calculated accordingly. Lockdown number 3 was not included as it was only 5 days. Total test numbers are not presented due to commercial issues. HPV was not analysed due to changes in testing requirements.

**Table 2:** Percentage difference of total pathology tests by year compared to 2019.

		State			
Test	Year	VIC (%)	NSW (%)	QLD (%)	WA/NT (%)
CBC	2020	−3.8	−3.3	−2.9	−1.1
	2021	2.3	−2.6	0.6	4.6
	2022	1.2	−2.7	−6.0	−1.6
	2023	6.4	−0.1	−4.6	8.5
	2024	7.7	1.1	−4.2	11.3
Histology	2020	−11.7	−2.2	−2.2	−16.6
	2021	−7.4	−6.8	−12	−9.1
	2022	−15.0	−9.8	−19.0	−11.6
	2023	−11.2	−6.4	−16.6	−3.4
	2024	−13.9	−4.0	−17.9	−2.9
PSA	2020	−2.7	−8.6	−5.1	−1.4
	2021	3.7	−4.4	1.9	7.8
	2022	3.5	−1.9	2.2	7.7
	2023	5.2	2.2	4.0	18.3
	2024	−15.6	−13.0	−12.2	11.3
HPV	2020	−41.4	−36.8	−33.7	−39.3
	2021	−47.0	−47.1	−44.7	−47.6
	2022	−52.5	−49.3	−52.2	−51.9
	2023	−34.5	−26.8	−31.5	−23.1
	2024	−27.3	−24.6	−29.1	−20.7
HbA1c (total) ^a^	2020	7.5	7.5	4.4	8.4
	2021	20.0	17.2	18.4	23.1
	2022	24.0	27.3	24.3	20.5
	2023	34.1	41.0	33.3	37.6
	2024	39.7	51.7	42.9	44.1
HbA1c (screening) ^c^	2020	0.4	11	11.7	7.7 ^b^
	2021	24.8	29.1	34.8	21.7 ^b^
	2022	47.6	48.0	40.8	25.8 ^b^
	2023	65.7	68.5	67.7	43.6 ^b^
	2024	88.5	94.4	90.6	59.0 ^b^
HbA1c (monitoring) ^d^	2020	−6.4	1.2	−0.1	−1.4 ^b^
	2021	−3.7	5.9	3	4.9 ^b^
	2022	−2.0	8.2	−6.6	−5.0 ^b^
	2023	−2.0	13.2	−0.8	−0.9 ^b^
	2024	−5.9	13.3	1.4	−2.0 ^b^
HIV ^e^	2020	−20.1	−17.8	−10.8	−7.5
	2021	−10.3	−14.3	−8	−0.9
	2022	−2.5	−11.7	−8.2	−9.1
	2023	4.5	−8.7	−3.5	1.8
	2024	3.5	−7.9	−5.7	0.2

^a^ Total refers to all HbA1C testing including screening, monitoring and other sources. ^b^ WA/NT HbA1c monitoring and screening values are based on data from April to December for each year. ^c^ Represents MBS item number 66841. ^d^ Represents MBS item number 66551. ^e^ Values do not include rapid tests. Total test numbers for each year are not presented due to commercial issues.

**Table 3:** Percentage difference in total rapid HIV tests in Australia by year compared to 2019.

	Year	% Difference
HIV rapid tests	2020	−29.2
	2021	−30.4
	2022	−20.8

## Data Availability

The datasets presented in this article are not readily available as they represent commercial data from a de-identified provider.

## Abbreviations

The following abbreviations are used in this manuscript:

COVID-19	SARS-CoV-2 coronavirus
CBC	complete blood count
GP	General Practice
HbA1c	haemoglobin A1c
HIV	human immunodeficiency virus
HPV	gynaecological cytology
MBS	Medicare Benefits Schedule
NSW	New South Wales (state)
PSA	prostate specific antigen
Qld	Queensland (state)
Vic	Victoria (state)
US	United States of America
WA/NT	Western Australia (state) and the Northern Territory
